# Pooled RNA sample reverse transcriptase real time PCR assay for SARS CoV-2 infection: A reliable, faster and economical method

**DOI:** 10.1371/journal.pone.0236859

**Published:** 2020-07-30

**Authors:** Ekta Gupta, Abhishek Padhi, Arvind Khodare, Reshu Agarwal, Krithiga Ramachandran, Vibha Mehta, Mousumi Kilikdar, Shantanu Dubey, Guresh Kumar, Shiv Kumar Sarin

**Affiliations:** 1 Department of Clinical Virology, Institute of Liver and Biliary Sciences, New Delhi, India; 2 Department of Hospital Operations, Institute of Liver and Biliary Sciences, New Delhi, India; 3 Department of Clinical Research, Institute of Liver and Biliary Sciences, New Delhi, India; 4 Department of Hepatology, Institute of Liver and Biliary Sciences, New Delhi, India; Rutgers University, UNITED STATES

## Abstract

**Background:**

Corona virus disease 2019 (COVID-19) which initially started as a cluster of pneumonia cases in the Wuhan city of China has now become a full-blown pandemic. Timely diagnosis of COVID-19 is the key in containing the pandemic and breaking the chain of transmission. In low- and middle-income countries availability of testing kits has become the major bottleneck in testing. Novel methods like pooling of samples are the need of the hour.

**Objective:**

We undertook this study to evaluate a novel protocol of pooling of RNA samples/elutes in performance of PCR for SARS CoV-2 virus.

**Study design:**

Extracted RNA samples were randomly placed in pools of 8 on a 96 well plate. Both individual RNA (ID) and pooled RNA RT-qPCR for the screening E gene were done in the same plate and the positivity for the E gene was seen.

**Results:**

The present study demonstrated that pool testing with RNA samples can easily detect even up to a single positive sample with Ct value as high as 38. The present study also showed that the results of pool testing is not affected by number of positive samples in a pool.

**Conclusion:**

Pooling of RNA samples can reduce the time and expense, and can help expand diagnostic capabilities, especially during constrained supply of reagents and PCR kits for the diagnosis of SARS-CoV-2 infection.

## Introduction

Corona virus disease 2019 (COVID-19) is a severe acute respiratory infection caused by the novel corona virus, severe acute respiratory syndrome coronavirus 2 (SARS-CoV-2) [1]. Initially started as cluster of cases from Wuhan, China [2] it has now spread to over 200 countries with 1,436,198 confirmed and 85, 522 deaths [3]. The laboratory diagnosis of SARS-CoV-2 is based primarily on nucleic acid amplification test (NAAT) like real time reverse transcriptase PCR (RT-qPCR). With an acute shortage of diagnostic kits, the present testing strategies mainly focus on testing the symptomatic individuals. But detecting the carrier or asymptomatic individuals holds the key in containing the spread of the infection into the community. Earlier the infected person is identified, sooner the spread of the infection can be contained and the surveillance machinery can be activated for contact tracing and ultimately break in the chain of transmission of the virus. Most of the countries have imposed lockdown to contain the infection but afterwards when the cases are expected to take a vertical trajectory, scaling up the testing would be a major challenge [4]. Innovative methods to conserve kits and reagents and human resources needs to be explored to enhance the testing. Pooling the diagnostic tests has been applied in other infectious diseases and is especially attractive as it requires no additional training, equipment, or materials. In this method, perfected over the years [5–7], samples are mixed and tested at a single pool, and subsequent individual tests are performed, only if the pool tests positive. We undertook this study to evaluate a novel protocol of pooling of RNA samples/elutes in performance of PCR for SARS CoV-2 virus.

## Methodology

### Sample collection

Combined nasopharyngeal and oropharyngeal swabs were collected by healthcare workers and transported in a 3 ml viral transport media(VTM) maintaining proper cold chain and sent to the virology laboratory of Institute of Liver and biliary sciences, New Delhi, India. A volume of 200 microlitre (μl) of the sample was further processed for viral nucleic acid extraction by Qiasymphony DSP Virus/ Pathogen mini kit (Qiagen GmbH, Germany) as per the manufacturers protocol in elutes of 60 μl each [8]. Each sample was subjected to the addition of 10 μl of spiked extraction control (EAC) at the time of extraction itself, to check the validity of the extraction procedure.

### Performance of RT-qPCR in the laboratory

The 5 μl of the extracted RNA elute/sample was subjected to RT-qPCR for the qualitative detection of SARS-CoV-2 RNA utilizing with AgPath-IDTM One-Step RT-PCR Reagents (Thermo Fisher Scientific) using a Applied biosystem (ABI) 7500 Real Time PCR system (Thermo Fisher Scientific) and LightMix^®^ SarbecoV E-gene (TIB MOLBIOL). Reactions were heated to 55°C for 5 minutes for reverse transcription, denatured in 95°C for 5 minutes and then 45 cycles of amplification were carried out for 95°C for 5 seconds and 60°C for 15 seconds using FAM parameter for E gene. This assay targets the detection of E gene for SARS as well as nCoV-2. The primer details are given in Table 1. All samples that were screened positive for E gene were confirmed by performance of RT-qPCR for the detection of specific RdRP gene of SARS-CoV-2 using LightMix^®^ Modular SARS-CoV-2 RdRP (TIB MOLBIOL) using similar PCR conditions as described above.

**Table 1:** Primers and probes for the RT-qPCR.

Gene	Oligonucleotide	Sequence
E gene	E_ Sarbeco _F	ACAGGTACGTTAATAGTTAATAGCGT
	E_Sarbeco_P1	FAM-ACACTAGCCATCCTTACTGCGCTTCG-BBQ
	E_Sarbeco_R	ATATTGCAGCAGTACGCACACA
RdRP gene	RdRp_SARSr-F	GTGARATGGTCATGTGTGGCGG
	RdRp_SARSr-P2	FAM-CAGGTGGAACCTCATCAGGAGATGC-BBQ
	RdRp_SARSr-R	CARATGTTAAASACACTATTAGCATA

FAM: 6-carboxyfluorescein; BBQ: blackberry quencher.

### Pooling of samples for the RT-qPCR

**Fig 1:**
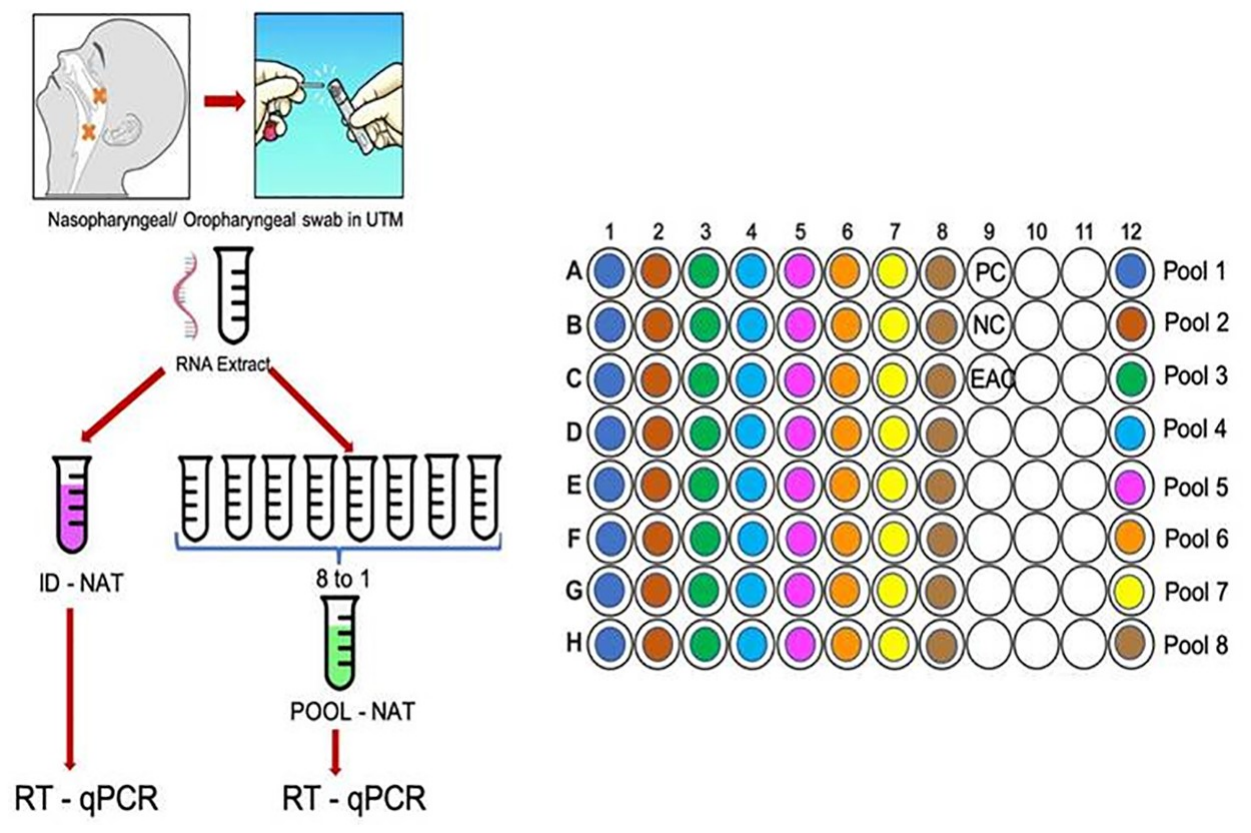
Scheme for pooling the samples on a 96 well plate.

Initially RNA samples that were obtained after extraction were randomly pooled into pools of 2, 4, 6, 8, 16 RNA elutes on a 96 well plate. The individual (ID) test and pool test were performed simultaneously and the results matched 100% in pools of 2, 4, 6 and 8 samples. In pool of 16 concordance between ID and pool results was not seen, hence it was decided to go ahead with the pooling of 8 RNA elutes (unpublished data). Subsequently, prospectively as RNA elutes were received in the laboratory, they were randomly pooled into pools of 8 RNA elutes on a 96 well plate as well as ID test as shown in Fig 1. Both ID and pooled RNA RT-qPCR for the screening E gene was done in the same plate (Fig 1). 5 μl of the RNA sample was taken for the PCR and pooling of 5 μl (8x5 = 40 μl) was done for the pooled test. After thorough mixing, 5 ul of the pooled RNA was taken for the pooled PCR. The volume of RNA sample was kept similar in both the ID as well as pooled PCR so that assay sensitivity is not affected. ID PCR and pooled PCR was performed in the same run, keeping the entire conditions uniform. Results were seen on the ABI software and each reaction was read for E gene after confirmation of the performance of EAC as well as positive control and negative control results. Ct value for each positive test was recorded and as per the WHO criteria, sample with Ct value ≤ 40 were considered as positive. All initial E gene positive were confirmed as positive if RdRP gene was also detected with Ct value ≤ 40.

### Statistical analysis

The data was compared for the ability of detection of E gene by ID test and pooled test using one sample paired t-test (Table 2). Data of Ct value is shown as mean ± standard deviations. The agreement between Ct values obtained for positive sample in one positive sample pool with ID test was done applying intra class correlation (ICC) followed by Bland and Altman graph. A P value of 0.05 or less was considered statistically significant.

**Table 2:** Comparison between the mean Ct values of individual test and pool test in different pool combinations.

Pool of 8 RNA samples		E gene detection			
Pool combinations	Total Number of Pools	Not detected in number of pools	Mean of ID test Ct value (± SD)	Mean of Pool test Ct value (± SD)	P value
1 positive + 7 negative	11	1	33.1 (±3.3)	35.6 (±3.3)	0.04
2 positive + 6 negative	5	0	30.4 (±3.7)	33(±3.1)	0.13
3 positive + 5 negative	5	0	34.2 (±3.7)	34.9(±2.6)	0.58
4 positive + 4 negative	1	0	31.7 (± 4.6)	31.7	-
8 negative	13	13	NA		-
**Total**	**35**	**14**			

Ct: cycle threshold, RNA: ribonucleic acid, ID: individual

### Ethics statement

The study was approved by the Institute Ethics Committee/Institutes review board of Institute of Liver and Biliary Sciences New Delhi, India. The study was conducted according to the principles expressed in the Declaration of Helsinki.(Ethics Approval number: IEC/2020/76/MA01). The patient informed consent was waived off by the Ethical committee as the research was done on the anonymised, de-identified RNA samples.

## Results

### Pool test vs ID test results

Out of 280 samples that were tested, 40 were positive and 240 were negative for SARS CoV-2 E gene with a positivity rate of 16.7% (95 CI; 12.2%-22.0%). All 40 were also positive for RdRP gene. Results were communicated to the patients as per the ID test results obtained. All the clinical details were de-identified, delinked and kept anonymised as per the Government of India data safety policy. Overall, 35 pools were made from these samples. On comparing the performance of pool test with ID test, concordance in performance was seen in 34 pools. Overall sensitivity of the pool test keeping ID test as gold standard was 95.4%, specificity 100%, positive predictive value 100% and negative predictive value of 92.86%. There were 13 pools where all the ID tests were negative and pool results were also negative. In 22 pools one or more positive sample was detected in the ID test (Table 2). Pools were further classified based on the number of positive samples present in them. There were 11 pools with 1 positive sample in each pool, 5 with 2 positive sample in each pool, 5 with 3 positive samples in each and 1 with 4 positive samples in it. 21 out of 22 (95.4%) pools could correctly identify the positive test. In 1 pool which had 1 positive and 7 negative sample, the ID test result showed delayed Ct at 39, therefore it was missed in pool testing. The sample was however positive for the RdRP gene and was given a positive result.

### Effect of pooling on the CT value of the positive result

In a positive test apart from getting a fluorescence curve the cycle number at which the fluorescence starts is also important, this is measured as Ct value. WHO has defined the criteria that any test which gives fluorescence after 40 cycles should not be considered as positive [9]. We wanted to see the effect of dilution on the Ct value of the positive result. Ct values of each positive sample in the ID test were compared with Ct values that were obtained in the pool test (Table 3). Overall, the mean CT value of ID was 32.68 while in pooled testing it was 34.24, only an increase in the Ct value of 1.56 very much within the reporting criteria of being called as positive. The pools with 1 sample positive and 2 samples positive in them, the ID test Ct values differed with the pool test Ct values but this difference was negligible when a pool had 3 or 4 positive samples in it. Still the Ct value rise after pooling was within the limit of being called as positive i.e < 40. This clearly shows that the dilution did not alter the Ct value of the positive result and a positive test will be reported as positive despite pooling.

**Table 3:** Comparison of individual test Ct value with pool test Ct value in the positive samples pool.

S. No.	Pool with 1 positive sample (n = 11)		Pool with 2 positive Sample (n = 5)		Pool with 3 positive Sample (n = 5)		Pool with 4 positive sample (n = 1)	
	ID Ct	Pool test Ct	ID Ct	Pool Ct	ID Ct	Pool Ct	ID Ct	Pool Ct
1.	29	32.13	28.9	32.3	39.82	34.09	31.72	31.72
2.	38	39	36.1		29.12		30.73	
3.	33.1	34.78	31.7	32.4	30.3		26.69	
4.	33.8	35.7	28.5		39.1	38.32	37.81	
5.	34.46	39.1	31.5	29.1	37.72			
6.	31.05	35.94	23.9		31.72			
7.	33.37	36.06	27.74	33.32	36.82	36.35		
8.	29.17	29.17	27.74		35.97			
9.	32.7	34.13	33.26	37.74	35.56			
10.	30.26	39.78	34.36		34.37	33.89		
11.	39.1	Negative			38			
12.					29.19			
13.					32.7	31.61		
14.					29.17			
15.					33.37			
**Mean**	**33.1**	**35.6**	**30.4**	**33.0**	**34.2**	**34.9**	**31.7**	**31.7**
**SD**	**3.3**	**3.3**	**3.7**	**3.1**	**3.7**	**2.6**	**4.6**	

Ct: cycle threshold, RNA: ribonucleic acid, ID: individual

### Effect of number of positive samples in a pool

**Fig 2:**
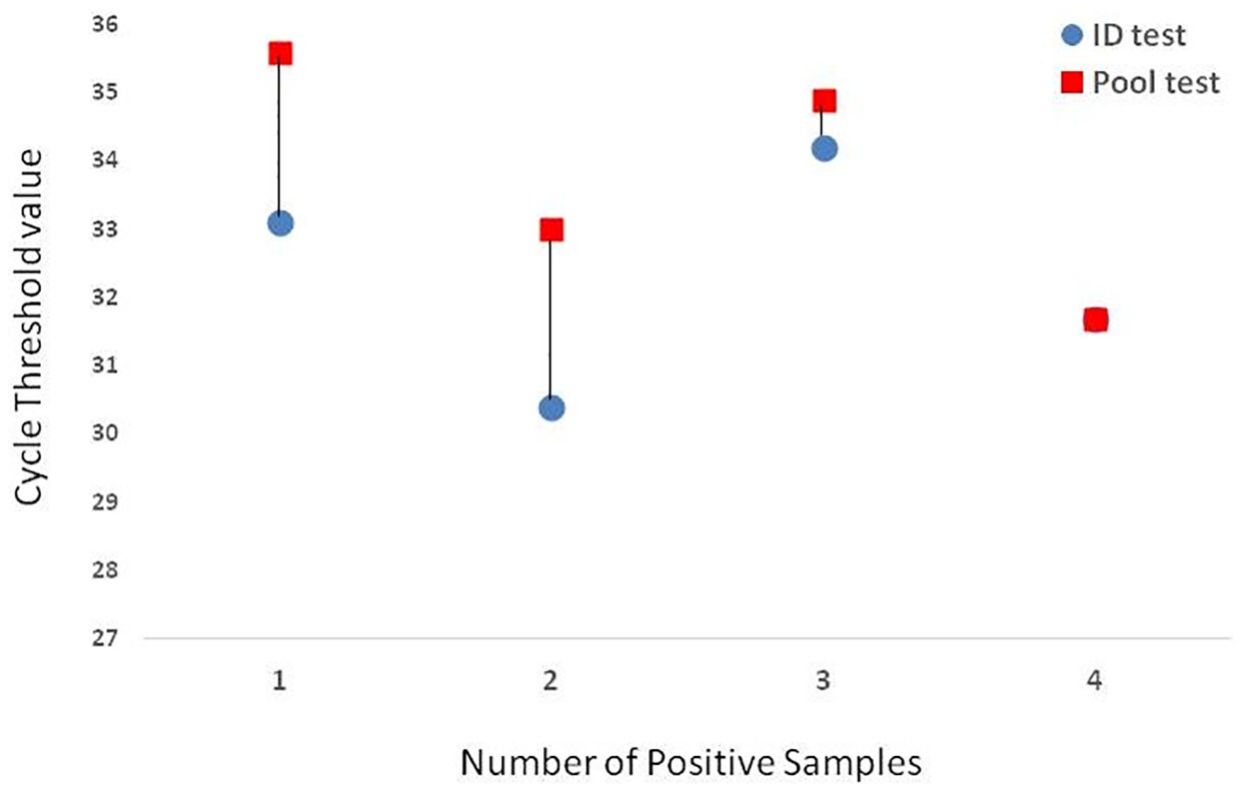
Effect of number of positive samples in a pool.

**Fig 3:**
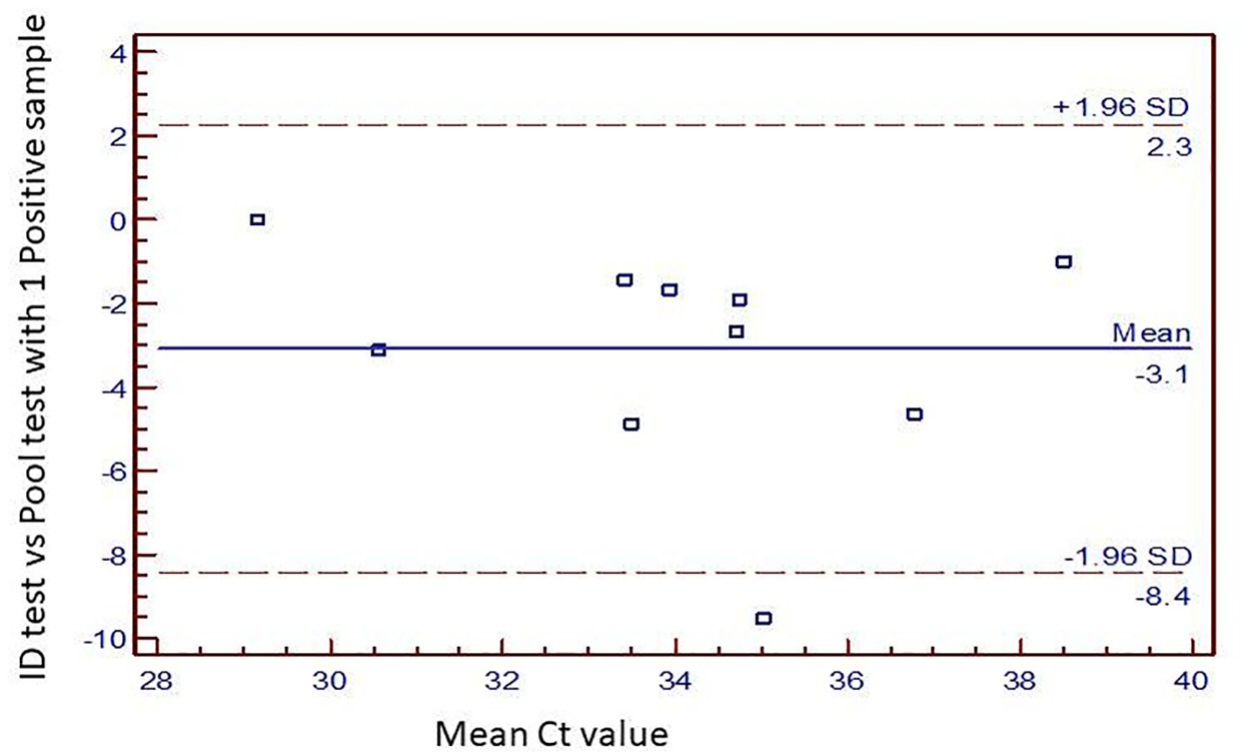
Performance of single positive sample pool.

We tried to look at the effect of presence of number of positive samples in a pool. As the number of positive samples in a pool increased the difference in the mean Ct value decreased (Fig 2). More the number of positives in a pool, more accurate the result of the pool testing was seen. When there were 4 positive samples in a pool, the mean ID Ct value were identical to mean pool Ct (Fig 2). On evaluating the performance of pools with only 1 positive sample in it, a bland and altman graph (Fig 3) showed an intra class agreement of 57.3% [(-29.4% to 88.7%), p = 0.024]. The reliability coefficient was found to be 74.5%. All tests were within 2 SD limit except 1. The limit of biasness was -8.4 to 2.3.

## Discussion

This novel study done prospectively demonstrates that pooling of RNA samples can be done easily in the laboratory. The present study also showed that the results of pool testing is not affected by number of positive samples in a pool. Pool testing can be easily applied for faster PCR results. Pooled testing has been used in many infectious diseases as a simple cost effective method to enhance the speed of diagnosis especially when large number of samples have to be tested during epidemic screening [10], pooling has been proven to work for RT-qPCR [7, 11].

In the present study we randomly pooled the RNA samples in pools of 8 based on the small preliminary data. However, when we did this study prospectively, we found that in 8 pool size, in 1 positive sample pool a single sample with very high Ct value was missed. So, smaller pool size than 8 would be even more effective (unpublished data). This being slightly different from another published study on pool testing with 1 positive sample pool where maximum number of pooling of samples was assessed retrospectively and in this study the authors found that pooling of 1 positive sample up to a pool of 64 samples with negative samples can be done without effecting the results [12]. Our study was different from this published study as we did the pooling prospectively, as and when the samples were received without knowing the results of the test and both pool test and ID test were done in the same PCR run. Therefore, in our study pooling in a real-life situation where number of positive samples in a pool cannot be predicted was studied. Further studies to determine single positive pool dilutions is underway in our laboratory. Number of positive samples in a pool could also influence the Ct value and final result of the test. We found that more than 1 positive samples in a pool did not affect the final result in pool testing, rather more the number of positive sample in a pool more accurate would be the results. Pooling of samples can be done at various levels, prior to RNA extraction, that is at the time of sample collection, putting the nasopharyngeal/ oropharyngeal swabs into a common viral transport media (VTM). By doing so the major bottle neck of RNA extraction can be removed. Pooling can also be done by pooling the VTMs and doing common extraction for pooled VTM samples, here the limitation is that in case a pool turns out to be positive, repeat sample collection or RNA extraction has to be done. This is time consuming and labour intensive. Cost of PCR compared to that of RNA extraction is at least 10 times. RNA extraction is usually done by automatic machine while plating up of a PCR test is mostly done manually. Thus, pooling of RNA samples before PCR is a faster and more economical method to do in a laboratory.

## Conclusion

As the COVID-19 pandemic spans across the globe, implementation of expanded testing in larger population groups is the only way out. We recommend that testing the pooled samples, if done properly, is reliable, and can take the fight of detecting COVID-19 to the next level at the earliest.

## Supporting information

S1 Data (XLSX)

S2 Data (XLSX)
